# Endoscopic lumbar sympathectomy as a treatment option for primary erythromelalgia - case report and review

**DOI:** 10.1590/1677-5449.202200952

**Published:** 2023-03-10

**Authors:** Marcelo de Paula Loureiro, Pietro Maran Novais, João Augusto Nocera Paulin, Daniel Benzecry de Almeida, Arlindo Nascimento de Lemos

**Affiliations:** 1 Universidade Positivo - UP, Curitiba, PR, Brasil.; 2 Instituto Jacques Perissat de Cirurgia Minimamente Invasiva - IJP, Curitiba, PR, Brasil.; 3 Instituto de Neurologia de Curitiba - INC, Curitiba, PR, Brasil.; 4 Faculdade de Medicina São Leopoldo Mandic - SLMANDIC, Campinas, SP, Brasil.

**Keywords:** lumbar sympathectomy, primary erythromelalgia, teenager, surgery, treatment, simpatectomia lombar, eritromelalgia primária, adolescente, cirurgia, tratamento

## Abstract

Erythromelalgia is a rare disease, involving pain, edema, redness, and hyperthermia in the limbs. It is extremely refractory to drugs, has no defined treatment, and causes psychological comorbidities in the patient. We describe a case of erythromelalgia involving a 17-year-old boy who had been suffering from the disease for almost 4 years prior to finding an effective treatment. A bilateral endoscopic lumbar sympathectomy was performed, limited to L2 and L3 resections. Four weeks after the procedure, the patient’s symptoms were significantly mitigated and at 8 months follow-up he remained almost asymptomatic. Endoscopic lumbar sympathectomy was an effective treatment for primary erythromelalgia in this teenager, with exceptional reduction of his symptoms.

## Resumo

Eritromelalgia é uma doença rara caracterizada por dor, edema, eritema e hipertermia nos membros. É extremamente refratária a medicamentos e não tem um tratamento definido, causando comorbidades psicológicas para o paciente. Descrevemos o caso de um menino de 17 anos que possuía eritromelalgia há quase 4 anos antes de ser submetido a um tratamento efetivo. Foi realizada simpatectomia lombar endoscópica bilateral limitada à ressecção dos gânglios L2 e L3. Após 4 semanas do procedimento, o paciente teve diminuição significativa dos seus sintomas e, com 8 meses de seguimento, permanece praticamente assintomático. A simpatectomia lombar endoscópica foi um tratamento eficaz para eritromelalgia primária em um adolescente, com redução excepcional dos seus sintomas.

## INTRODUCTION

Erythromelalgia (EM) is an extremely rare disease and is especially rare in kids. It involves episodes of erythema and burning pain in the limbs that can be extremely bothersome. The prevalence is unknown, but it is predominant among girls and it usually appears during adolescence. It is divided into 2 types: primary and secondary. The secondary type has an underlying cause and is rarer in children. The primary type may have an inheritable cause, but in most cases the cause is unknown.^1^

## CASE DESCRIPTION

**Figure 1:**
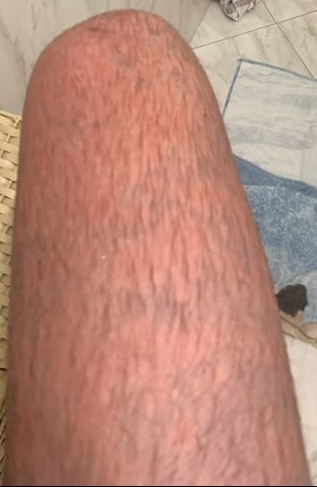
Appearance of erythromelalgia in thigh.

**Figure 2:**
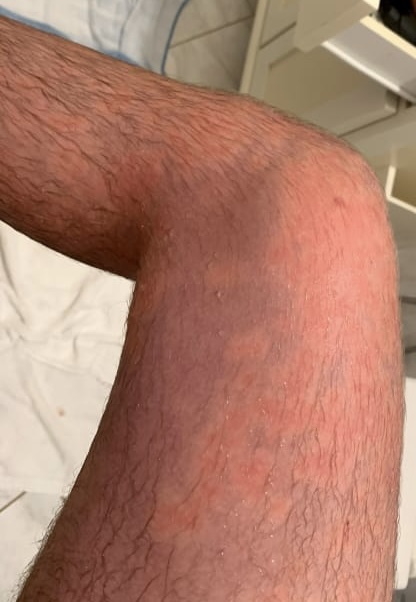
Appearance of erythromelalgia in the right thigh.

**Figure 3:**
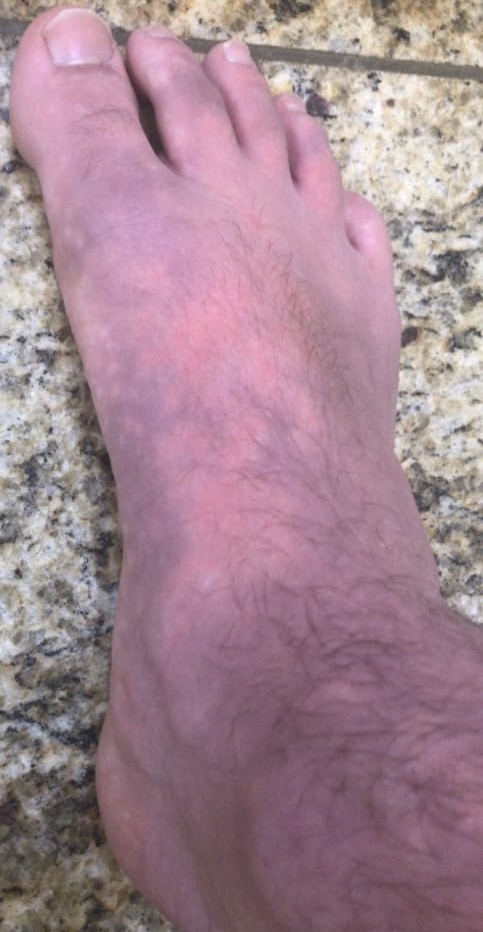
Appearance of erythromelalgia in the right foot.

**Figure 4:**
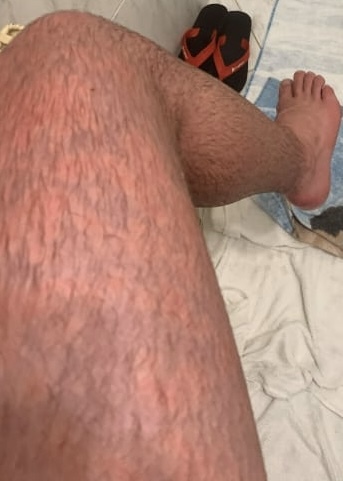
Appearance of erythromelalgia in the left thigh.

**Figure 5:**
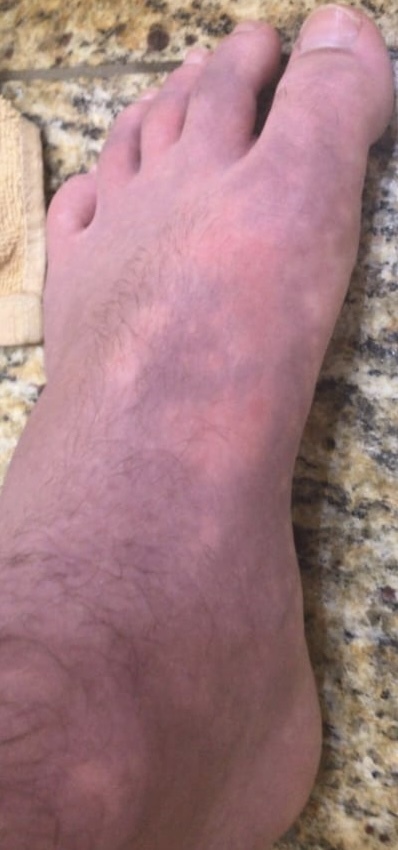
Appearance of erythromelalgia in the left foot.

A 17-year-old boy came to us seeking surgical treatment of primary erythromelalgia as a last resort, after several attempts to treat this unusual disease. Initially, when he was 14 years old, he had noticed bilateral allodynia of the ankles, assessed at level 3-4 on a verbal numerical rating scale (VNRS). A first evaluation with an orthopedist detected edema and discoloration extending from the feet to the inferior third of the thighs (Figure 1, 2, 3, 4,5). He was therefore referred to a rheumatologist. Autoimmune antibodies were investigated, with negative results, and laboratory tests were also normal. His condition gradually worsened and episodes were triggered by exercise and heat. The edema persisted and he started to have erythema, local hyperthermia, and compression pain, described as like “tight pants”.

Primary EM was diagnosed based on the clinical history of pain, triggers, full body thermography, and a negative genetic mutation test for SCN9A.

Progressively, the disease was profoundly affecting his quality of life. Pain could only be relieved by lying down, elevating his legs, and setting the air-conditioner to 18º C.

He tried different treatments, including consulting with a neurosurgeon specialized in pain relief and a psychiatrist. Pregabalin was used for 5 months at 75 mg twice a day, with no effect. At that point his VNRS score had worsened, reaching 5-6. Several other medications were also prescribed, including antidepressants, acetaminophen 300 mg twice a day, and tramadol 30 mg twice a day for 5 months, with almost no change in the general picture, but additional side effects. Episodes became constant, affecting his sleep and social life.

One year after the diagnosis of EM, a combination of venlafaxine 250 mg/day and tramadol 100 mg twice a day was finally able to reduce the pain intensity score from 8 to 4 and give mood support, stabilizing his condition. This allowed him to return to some social activities and the treatment was maintained for 2 years.

This afforded considerable relief, but the condition was still causing pain, so the family decided to seek a different approach. They consulted our team to try to achieve better control of the EM. We explained that endoscopic lumbar sympathectomy (ELS) could sometimes be used as a last resort for his condition and that if we could achieve 50% pain relief, that would be a tremendously positive result and could possibly help him lower his medication dosages and reduce their side effects. Four years after the patient’s first episode, his family agreed to surgery and he underwent bilateral ELS.

### Surgical technique

The patient underwent general anesthesia with endotracheal intubation and was placed in a dorsal decubitus position, slightly lateralized, with a cushion under the lumbar region. Retroperitoneal access was obtained using an intraperitoneal view to guide retroperitoneal placement of the first trocar halfway between the iliac crest and the inferior costal facet on each side, at the median axillary line.

Once the 10 mm scope had been positioned, the retroperitoneal space was dissected, using the optics initially. After the extraperitoneal space was established, the other trocars were placed equidistant to the first one in a semi-lunar distribution. Then, two 3-mm ports were inserted under direct view and the space was further developed. The transversalis fascia was dissected, reaching the psoas muscle, the main landmark in this space. Care was taken to keep in contact with the psoas, avoiding the wrong plane of the quadratus lumborum. The genitofemoral nerve was identified in a medial direction, above the psoas tendon. The ureter was then located and isolated in the direction of the peritoneum. Dissection continued until the lumbar vertebra. Just before reaching it, on the left side, we found the aorta and the iliac arteries, and on the right side, the vena cava. Following this, the lumbar sympathetic trunk was identified medially to the muscle, where it is covered by a capsule formed by the insertion of the psoas muscle at the lumbar spine.

As the capsule was opened, the trunk and its ganglia were revealed. After correct identification, we proceeded with careful resection of the sympathetic ganglia - the second and third lumbar ganglia. The chain removed was 4 cm in length. The entire surgical procedure lasted around 72 min, with no complications.

After discharge, the patient was reevaluated after 4 weeks, 1 month, 4 months, and 10 months. The first 3 weeks showed no great improvement in his condition. Edema was still present, although control of pain relief was slightly better. After 4 weeks, the symptoms started decreasing and at 4 months he reported a 95% reduction in his symptoms. Today, 10 months after ELS, he rates his pain as level 1 and has no other symptoms, enabling a full return to an active and social life. He has weaned off venlafaxine and has stopped taking tramadol and antidepressants. Besides compensatory sweating in his torso and anhidrosis in his feet, he has had no other side effects of ELS.

The project was approved by the research ethics committee at Universidade Positivo, protocol number 5.659.788.

## DISCUSSION

Erythromelalgia is a rare disease. The classic symptoms of EM are redness, pain, increased temperature, and swelling, usually triggered by heat or physical exercises.^1^^,^^2^ An initial allodynia can be observed in some cases.^3^^,^^4^ These episodes may be intermittent (81% of patients) or constant (19% of patients).^1^ A retrospective study published by Cook-Norris et al.^5^ showed that all patients had involvement of the feet and 50% also had upper extremity involvement, being symmetric in the primary form. The importance and severity of EM comes from its high morbidity, both physical and psychological. This is unfortunately demonstrated by its frequent association with suicide.^1^^,^^4^

The physiopathology of primary EM is not very clear, but theories regarding abnormal vascular dynamics have been raised. Accordingly, the precapillary sphincters located in the skin of feet and hands would be constricted in EM, reducing nutritional flow, whereas anatomical vascular shunts would be opened, allowing for high blood flow, causing the characteristic symptoms of EM, hypoxia, and tissue damage.^2^^,^^3^^,^^4^^,^^6^^,^^7^ However, full blood investigations should be done regularly, since EM may occur years prior to a myeloproliferative disorder.^1^^,^^2^

There is no specific treatment for EM and patients have variable responses to the pharmacological and surgical treatments that are available.^1^^,^^3^ Antidepressants, anticonvulsants, and anti-inflammatories can be used, but real benefits are seen in less than half of the patients.^1^ In case of failure of these approaches, surgical options should be considered.^8^

Lumbar sympathectomy (LS) is a surgical procedure in which part of the sympathetic trunk and ganglia are irreversibly damaged, most commonly L2-L4. This interrupts the efferent signals to α-1 receptors, causing relative vasodilation in small vessels of the lower limbs. Also, exocrine glands and nociceptors lose their efferent and afferent signals, respectively, reducing sweating and perception of pain. Lumbar sympathectomy is indicated in severe cases of ischemic lower limb disease, hyperhidrosis, and pain. It can be performed in various ways. The endoscopic approach has more benefits and fewer complications for the patient.^9^^,^^10^^,^^11^ Nevertheless, side effects and complications can still occur with this treatment. Compensatory sweating, genitofemoral neuritis, and sexual dysfunction can occur.^9^^,^^12^ The evaluation of the post-operative benefits depends on the indications for surgery.^9^^,^^11^^,^^13^^,^^14^^,^^15^

The indication of LS for EM is not well-defined because of the small number of cases, but it has been performed to treat EM since the last century.^15^^,^^16^ A study recruiting 13 patients for chemical LS to treat EM reported that 9 patients maintained complete response after 6 months and 4 patients were still feeling well after 6 years. Pain decreased in all 13 patients, comparing before treatment and 1 day after the surgery.^17^ In 3 case reports of thoracic sympathectomy as treatment for primary EM in adult women, all patients had notable relief from symptoms, with no complications.^18^^,^^19^

The initial edema after the operation is consistent with vasodilation caused by the sympathectomy. However, 4 months after surgery his condition had improved remarkably. The collateral effects were expected of ELS and the patient reported a reminiscent memory of the pain. This shows how striking the EM was to him. The results of ELS helped his mental health to improve outstandingly, enabling him to return to normal activities.

## CONCLUSION

Endoscopic lumbar sympathectomy was used successfully in a teenager as treatment for primary erythromelalgia, with remission of symptoms and a remarkable improvement in his quality of life.
